# Fine mapping and candidate gene analysis of gynoecy trait in chieh-qua (*Benincasa hispida* Cogn. var. *chieh-qua* How)

**DOI:** 10.3389/fpls.2023.1158735

**Published:** 2023-04-20

**Authors:** Min Wang, Songguang Yang, Wei Liu, Zhenqiang Cao, Lin Chen, Wenrui Liu, Dasen Xie, Jinqiang Yan, Biao Jiang, Qingwu Peng

**Affiliations:** ^1^ Vegetable Research Institute, Guangdong Academy of Agricultural Sciences, Guangzhou, China; ^2^ Guangdong Key Laboratory for New Technology Research of Vegetables, Guangdong Academy of Agricultural Sciences, Guangzhou, China

**Keywords:** chieh-qua, gynoecy, gene mapping, *CqNET4*, ethylene synthesis related genes

## Abstract

Gynoecy demonstrates an earlier production of hybrids and a higher yield and improves the efficiency of hybrid seed production. Therefore, the utilization of gynoecy is beneficial for the genetic breeding of chieh-qua. However, little knowledge of gynoecious-related genes in chieh-qua has been reported until now. Here, we used an F_2_ population from the cross between the gynoecious line ‘A36’ and the monoecious line ‘SX’ for genetic mapping and revealed that chieh-qua gynoecy was regulated by a single recessive gene. We fine-mapped it into a 530-kb region flanked by the markers Indel-3 and KASP145 on Chr.8, which harbors eight candidate genes. One of the candidate genes, *Bhi08G000345*, encoding networked protein 4 (*CqNET4*), contained a non-synonymous SNP resulting in the amino acid substitution of isoleucine (ATA; I) to methionine (ATG; M). *CqNET4* was prominently expressed in the female flower, and only three genes related to ethylene synthesis were significantly expressed between ‘A36’ and ‘SX.’ The results presented here provide support for the *CqNET4* as the most likely candidate gene for chieh-qua gynoecy, which differed from the reported gynoecious genes.

## Introduction

Sex differentiation in higher plants is often determined by sex chromosomes and sex-determining genes (Lai et al., 2017), and the regulation of unisexual flower development has been the focus of plant sex determination (Bai et al., 2004). According to the distribution or proportion of three flower types (female flowers, male flowers, and complete flowers) in a plant, the cucurbit plant can be classified into six phenotypes, namely, gynoecy, monoecy, subgynoecy, androecy, andromonoecy, and hermaphrodites (Li et al., 2019). Gynoecy has been the focus of research on the sexual type of cucurbits as it directly affects the yield and hybrid seed purity (Boualem et al., 2009; Boualem et al., 2015; Zhang et al., 2021). Melon and cucumber have been excellent models for studying the molecular mechanisms of gynoecism development. The recessive gene *CmWIP1* (encoding the C2H2-type zinc finger protein transcription factor) (Boualem et al., 2015) and ethylene synthesis-related genes (Zhang et al., 2021) are involved in gynoecism regulation.

The expression of the *CmWIP1* gene in melon could inhibit carpel development and the expression of downstream *CmACS7*, thus promoting the development of the male flower (Martin et al., 2009; Boualem et al., 2015; Boualem et al., 2016). Gene editing of *CsWIP1* in cucumber exerts a gynoecious phenotype (Chen et al., 2016; Hu et al., 2017). The chromosomal translocation of the *ClWIP1* gene in watermelon leads to an insertion mutation and results in gynoecy (Zhang et al., 2020). A recent study demonstrates that CmWIP1 in melon interacts with the co-suppressor protein TPL and binds to the promoter of CRC (carpel development gene *CRABS CLAW*), which deacetylates the CRC genomic protein to suppress its expression, finally interfering with the floral meristem determination in the carpel primordium and promoting the male flower development. However, mutation of *CmWIP1* can promote the expression of CRC and also promote the development of the carpel and the expression of the *CmACS7* gene, thus producing female flowers (Zhang et al., 2021).

Except for the negative regulation of the *CsWIP1* gene in cucumber, there is also a dominant *F* gene. A recent study identifies that the *F* gene is *ACS1G* rather than the MYB transcription factor in the repeat sequence. Due to the structural variation of the genome, *ACS1G* acquired a promoter and a new expression pattern, which is different from that of *ACS1* in monoecious materials (Li et al., 2020; Zhang et al., 2021). In addition, *CsACS1G* acts upstream of *CsWIP1* and inhibits its expression by producing ethylene, resulting in a gynoecy phenotype (Zhang et al., 2021).

Chieh-qua (*Benincasa hispida* Cogn. var. *chieh-qua* How), a wax gourd variety, is an important fruit vegetable widely cultivated in South China and Southeast Asia that is rich in vitamins, propanol diacid, and other nutrients (He et al., 2007). Like other cucurbit crops, chieh-qua also has several sexual flower types including gynoecism, which is important in breeding and hybrid seed production. However, the genetic studies on gynoecy in chieh-qua are very limited. Since the release of the reference genome of wax gourd (Xie et al., 2019), it has provided us with a great opportunity to explore the genetic basis of chieh-qua, such as the trait of gynoecy.

In this study, we fine-mapped the gynoecy trait using an F_2_ population derived from the cross between ‘A36’ and ‘SX.’ Candidate genes in the 530-kb interval were sequenced and further assessed by the qRT-PCR assay. Combining sequencing and expression analysis, we found that the candidate gene related to chieh-qua gynoecy was different from the homologous gynoecious genes *WIP1* and *ACS1G*. Collectively, our findings not only provided practical markers for molecular gynoecious breeding of chieh-qua but also enriched the cucurbit sex differentiation theory.

## Materials and methods

### Plant materials

Two chieh-qua inbred lines, ‘A36’ and ‘SX,’ were used for phenotypic characterization and segregating populations. ‘A36’ with a gynoecious phenotype was a homozygous inbred line derived from the native variety ‘Jiangxinjie’ in Guangdong Province. ‘SX’ with a monoecious trait was also a high-generation inbred line, derived from a cross of ‘Cuiyu’ and ‘Guiyou’ chieh-qua. F_1_ plants were obtained by crossing ‘A36’ (P_1_) and ‘SX’ (P_2_), and the F_2_ population was obtained by self-crossing F_1_ plants. Parents and F_1_ individuals were planted in the spring of 2020. A total of 308 and 196 F_2_ individuals were planted in the spring and autumn of 2020, respectively, and a total of 2,120 F_2_ individuals were planted in the autumn of 2021. For phenotyping, individuals producing only female flowers in the main stem were considered gynoecious, while individuals producing both male and female flowers were monoecious. All plants were grown in the Baiyun study base of Guangdong Academy of Agricultural Sciences (Guangzhou, China) at 23.4°N (latitude) and 113.4°E (longitude).

### Generation of the whole genome resequencing data

Young leaves of parents and F_2_ individuals were used to extract genomic DNAs using cetyltrimethylammonium bromide (CTAB) (Healey et al., 2014). We constructed the gynoecium pool (G-pool) and the monoecism pool (M-pool) by mixing an equal amount of DNAs from 30 gynoecious and 30 monecious F_2_ plants, respectively. Then, the DNA of parents and two DNA pools were sequenced using the Illumina sequencing platform by Genedenovo Biotechnology Co., Ltd. (Guangzhou, China). To ensure the correctness of the resequencing results, the depth of sequencing data in the parent pool was larger than 10×, and that in the G-bulk and M-bulk was larger than 50×.

### Alignment and analysis of BSA data

In order to align clean reads from each sample, we used the software Burrows-Wheeler Aligner (BWA) (Li and Durbin, 2010; Abuín et al., 2015) against the public reference genome (Xie et al., 2019), and alignment files were converted to SAM/BAM files using SAMtools (Li et al., 2009). Variant calling was carried out for all the samples using GATK’s UnifiedGenotyper (McKenna et al., 2010). Single-nucleotide polymorphisms (SNPs) and indels were filtered using GATK’s Variant Filtration with proper standards (-Window 4, -filter “QD < 4.0||FS > 60.0|| MQ < 40.0”, -G_filter “GQ < 20”). Next, to determine the physical positions of each obtained variant, we used the software tool ANNOVAR (Wang et al., 2010a) to align and annotate SNPs or indels, and the SNP index and delta (SNP index) were calculated and analyzed to identify the candidate region for gynoecium in chieh-qua (Takagi et al., 2013; Lu et al., 2014). Furthermore,the original sequencing data are being uploaded to the NCBI database with the BioProject ID:PRJNA941947.

### Development of molecular markers for fine mapping

Based on the primary mapping of BSA-seq, simple sequence repeat (SSR) markers were used for gene linkage analysis. New indel and Kompetitive Allele-Specific PCR (KASP) markers were developed based on the DNA sequence polymorphisms from the parents’ resequencing data and B227 reference genome (http://cucurbitgenomics.org/organism/22). The primer design was performed using the Primer Premier 5 (Premier Biosoft, Palo Alto, CA, USA). All primers were synthesized commercially, and all newly developed markers were firstly screened for polymorphism between the two parental lines. The primer information is listed in **Supplemental Table 1**. The obtained polymorphic markers were applied to the 2,120 F_2_ individuals. Based on the confirmed recombination events and sex phenotype of the detected recombinants, the initial mapping region was further narrowed down.

### Amplification of candidate genes

Total RNA was extracted from the terminal buds of A36 and SX. The cDNA was synthesized from total RNA using the PrimeScript RT reagent kit and gDNA Eraser (TaKaRa, Dalian, China) according to the manufacturer’s instructions. The coding sequences of eight candidate genes (*Bhi08G000338*–*Bhi08G000345*) in the interval were amplified using the PCR primers listed in **Table S2**. The PCR amplifications were then performed using the PrimeSTAR^®^ Max DNA Polymerase (Takara Bio, Shiga, Japan) and respectively ligated into pEASY^®^-Blunt Zero Cloning Vector (TransGen Biotech, Beijing, China) according to the manufacturer’s instructions. We identified the positive clones using colony PCR and analyzed the Sanger sequencing results using DNAMAN software to confirm the candidate gene.

### Phylogenetic analysis

In order to analyze the evolutionary relationship between CqNET4 and its homologs, we performed a Basic Local Alignment Search Tool (BLAST) (https://blast.ncbi.nlm.nih.gov/Blast.cgi) to explore the homolog sequences from the NCBI database using the CqNET4 protein sequence as the query. The obtained amino acid sequences were aligned using ClustalW, and a phylogenetic tree was built using the neighbor-joining method with 1,000 bootstrap replications in the MEGA X software.

### Subcellular localization

The full-length coding sequence (CDS) of CqNET4 was cloned into pCAMBIA1305 vector to create a 35S:GFP-CqNET4 fusion construct, which was subsequently transformed into *Agrobacterium tumefaciens* strain EHA105. The constructs were infiltrated into *Nicotiana benthamiana* leaves, and the mesophyll protoplasts were obtained by polyethylene glycol-mediated infiltration. Fluorescence signals were visualized using an LSM880 laser scanning confocal microscope (Carl Zeiss, Jena, Germany).

### qRT-PCR analysis

RNA was prepared according to the instructions of the TRIzol^®^ reagent (TaKaRa, Japan) and purified and concentrated using an RNeasy MinElute Cleanup Kit (Qiagen, Germany). Quantitative real-time PCR (qRT-PCR) (20 μl) was performed with 0.5 μl of cDNA, 0.2 μM of primer mix, and SYBR Premix Ex Taq Kit (TaKaRa, Japan). We used the 7500 Real-Time PCR System (Applied Biosystems, America) to monitor the gene expression by fluorescence at the end of each cycle. For each plate, three technical replicates and three biological replicates were generated. The candidate gene expression was evaluated by the 2^−ΔΔCt^ method (Livak and Schmittgen, 2001). The significant differences were detected by IBM SPSS Statistics 20 (Student’s *t*-test). The primer sequences are listed in **Supplemental Table 3**.

## Results

### Genetic inheritance of gynoecious trait

**Figure 1:**
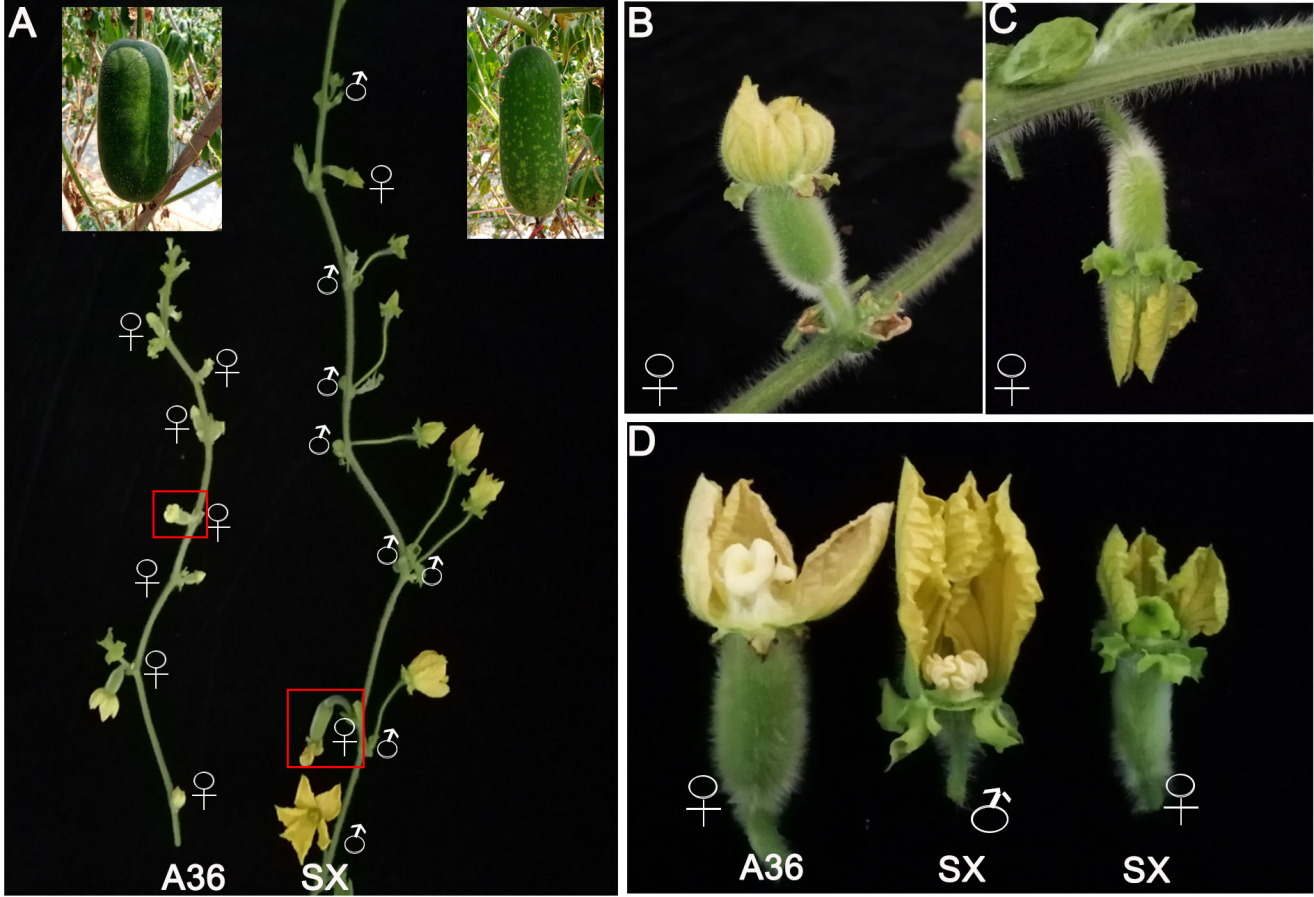
Characteristics of chieh-qua flower types. **(A)** The typical images of flower types and fruits of A36 and SX. **(B, C)** The female flowers of gynoecious (A36) and monoecious (SX) lines. **(D)** The stigma color of A36 and SX female flowers. ♀ represents a female flower; ♂ represents a male flower.

To determine the inheritance pattern of the gynoecious trait, the gynoecious parent A36 was crossed to the monoecious parent SX to construct a segregating population. When the individual plants of parents and the F_1_ and F_2_ populations start to bear flowers, the sexual flower type (female or male flower) on every node was determined. The images of typical flower types for the two parental lines are shown in **Figure 1**. Generally, every node in the main stem of A36 was a female flower, whereas both female and male flowers existed in the SX main stem (**Figure 1A**). Furthermore, the color of the stigma of female flowers differed, with A36 having white stigmas and SX having green stigmas (**Figures 1B–D**). Like the phenotype of SX, all F_1_ individuals produced both female and male flowers. The segregation in the F_2_ population was 235 plants with monoecious phenotype and 73 with gynoecious phenotype (*χ* ^2^_3:1_ = 0.28; *P* = 0.6) in the spring of 2020 and 144 monoecious plants and 52 gynoecious plants (*χ* ^2^_3:1_ = 0.24; *P* = 0.62) in the autumn of 2020 (**Table 1**), indicating that the chieh-qua gynoecious trait was caused by a single recessive gene.

**Table 1:** Genetic analysis of chieh-qua gynoecy using the F_2_ population derived from A36 and SX.

Materials	Number of total plants	Monoecious plants	Gynoecious plants	Expected ratio	*P*
A36	20	0	20	–	–
SX	20	20	0	–	–
F_1_	20	20	0	–	–
F_2_ (2020 spring)	308	235	73	3:1	0.6
F_2_ (2020 autumn)	196	144	52	3:1	0.62

### Fine mapping of the candidate gene

**Figure 2:**
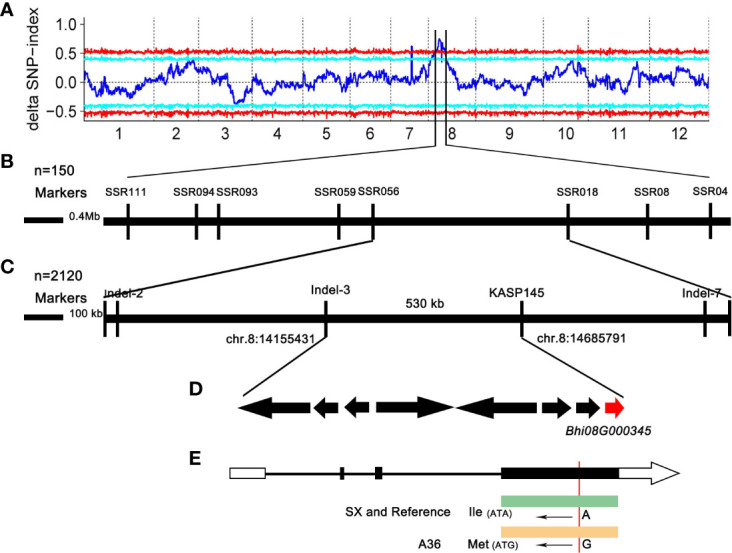
Genetic mapping of the gynoecious gene in chieh-qua. **(A)** Delta SNP index distribution on chieh-qua chromosomes. The abscissa is the chromosome name, the blue line represents the fitted ED value, and the red line represents the significance association threshold of ±0.5. The higher the ED value, the better the association effect of this point. **(B, C)** Fine mapping of the gynoecious gene. **(D)** Candidate genes in the fine mapping region. **(E)** The candidate gene structure and comparison of the base sequence in ‘A36’ and ‘SX.’ The white boxes, black boxes, and solid line represent 5′ and 3′ UTR, exons, and introns, respectively.

To determine the chromosomal location of the gene controlling the gynoecious trait, DNA pools from 30 homozygous gynoecious (G-pool) and 30 homozygous monoecious (M-pool) F_2_ plants were created and genotyped using the Illumina high-throughput sequencing platform. We finally obtained 207,126,558 and 198,796,506 reads from the G-pool and the M-pool, respectively. These reads were aligned to the B227 reference genome (Xie et al., 2019), and the average paired mapped reads were 175,114,378 with 93.8% align ratio (**Supplemental Table 4**). Next, the SNP index graphs of the G-pool and the M-pool (**Supplemental Figure 1**) were constructed by computing the average SNP index, and the delta SNP index was computed and plotted against the genome locations based on the information of the SNP index of these two extreme pools. The results showed that the region on Chr.8 from 13.08 to 16.06 Mb, with a total length of 2.98 Mb (**Supplemental Figure 1**; **Figure 2A**), was the most likely interval, indicating the presence of a significant locus governing the related gynoecy characteristic.

Based on the reference genome, we designed 45 SSR markers in the candidate region for screening the polymorphism between A36 and SX. In the candidate interval, eight polymorphic SSR markers were identified and mapped. Combining the linkage analysis of 150 F_2_ individuals revealed that SSR056 and SSR018 were the two closest flanking markers with 1.5 Mb physical length (**Figure 2B**). In order to fine map the candidate locus, 2,120 F_2_ plants were firstly screened using the major flanking SSR056 and SSR018 markers to obtain 25 recombinant plants. In addition, 20 indel and 18 KASP markers were further developed to genotype the recombinant plants. After genotyping the F_2_ plants, only three indel and one KASP markers were polymorphic with a low polymorphism rate. Finally, the candidate gene was positioned between 14.15 and 14.68Mb in an approximately 530-kb region bordered by the markers Indel-3 and KASP145 (**Figure 2C**).

According to the reference genome sequence (http://cucurbitgenomics.org/organism/22), the genomic candidate area contained eight protein-coding genes (*Bhi08G000338*–*Bhi08G000345*) (**Figure 2D**), which encoded the MYB transcription factor, secretory protein HlyD family protein, NETWORKED 4 (NET4) protein, and so on (**Figure 2D**; **Table 2**). In order to compare the eight annotated genes in the candidate interval, these candidate genes from parents were amplified by PCR. Sequencing data showed that except *Bhi08G000345*, CDS sequence alignment showed no nucleotide differences in the other genes between ‘A36’ and ‘SX,’ indicating that the other seven genes were not associated with gynoecy. Indeed, sequence analysis of *Bhi08G000345* exerted an SNP base substitution (A→G) in the third exon, causing an L (SX: Lle) to M (A36: Met) amino acid alternation (**Figure 2E**). Moreover, we found that *Bhi08G000345* shared a sequence homology with *Arabidopsis thaliana* (*AT5G58320*), which encoded the protein NETWORKED 4. Therefore, *Bhi08G000345* (denoted *CqNET4*) is the most possible gene governing gynoecy in chieh-qua.

**Table 2:** The candidate genes in the mapping interval.

Gene ID	Start	End	Ortholog in *Arabidopsis thaliana*	Annotation
Bhi08G000338	14,125,452	14,127,295	AT1G68320	MYB transcription factor
Bhi08G000339	14,159,456	14,171,871	–	Unknown protein
Bhi08G000340	14,207,969	14,208,250	–	Secretion protein HlyD family protein
Bhi08G000341	14,213,402	14,214,838	AT2G28690	TOX high mobility group box family member 4-A, putative isoform 4
Bhi08G000342	14,412,611	14,446,386	AT1G55750	General transcription factor IIH subunit 1
Bhi08G000343	14,479,785	14,541,147	AT1G06840	Protein kinase family protein
Bhi08G000344	14,585,154	14,599,049	AT5G20920	Eukaryotic translation initiation factor 2
Bhi08G000345	14,599,610	14,606,100	AT5G58320	Protein NETWORKED 4A

### Development of molecular markers

**Figure 3:**
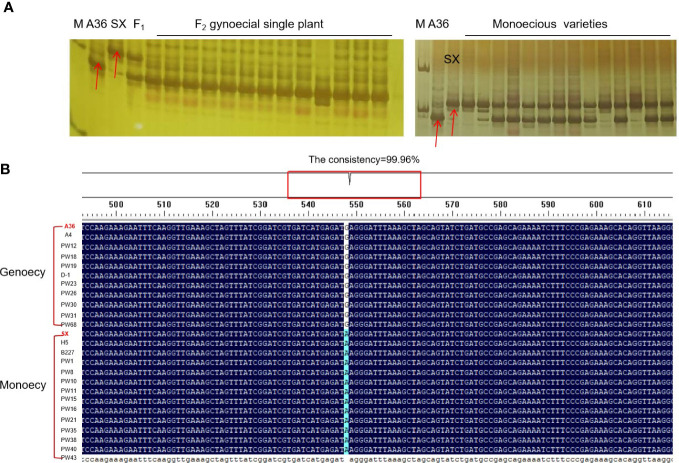
Validation of molecular markers closely linked to gynoecy trait. **(A)** The detection of Indel-3 in F_2_ individuals and monoecious plant. **(B)** Analysis of variation of *CqNET4* in 25 chieh-qua materials.

In order to identify the molecular markers linked to chieh-qua gynoecy, the DNA of 13 F_2_ plants and 10 monoecious varieties was amplified using Indel-3 primers. We found that the accuracy of the marker was over 93% (**Figure 3A**). Next, we used the KASP145 marker to analyze the DNA of 50 germplasm chieh-qua materials consisting of 5 gynoecious plants and 45 monoecious plants. The genotype of gynoecy was T:T, the monoecious genotype was C:C, and the heterozygous genotype was T:C. Results showed that except for the phenotypes of two materials (AW2 and AW10) inconsistent with the genotypes, the detection accuracy of KASP145 was 96.0% (**Table 3**). In addition, we analyzed the variation of CqNET4 in 25 chieh-qua materials and found that base 548 of the exon region of this gene was ‘G’ in the gynoecious materials, while ‘A’ was found in the monoecious materials (**Figure 3B**).

**Table 3:** Identification of KASP145 in 50 chieh-qua germplasm resources.

ID	Name	Phenotype	Genotype	ID	Name	Phenotype	Genotype
1	AW1	Gynoecy	T:T	26	AW25	Monoecy	T:C
2	AW3	Gynoecy	T:T	27	AW26	Monoecy	T:C
3	AW45	Gynoecy	T:T	28	AW27	Monoecy	T:C
4	AW15	Gynoecy	T:T	29	AW28	Monoecy	T:C
5	AW10	Gynoecy	C:C	30	AW29	Monoecy	T:C
6	AW2	Monoecy	T:T	31	AW30	Monoecy	T:C
7	AW4	Monoecy	T:C	32	AW31	Monoecy	T:C
8	AW5	Monoecy	C:C	33	AW32	Monoecy	T:C
9	AW6	Monoecy	C:C	34	AW33	Monoecy	T:C
10	AW7	Monoecy	T:C	35	AW34	Monoecy	C:C
11	AW8	Monoecy	C:C	36	AW35	Monoecy	T:C
12	AW9	Monoecy	T:C	37	AW36	Monoecy	T:C
13	AW11	Monoecy	T:C	38	AW37	Monoecy	T:C
14	AW12	Monoecy	T:C	39	AW38	Monoecy	T:C
15	AW13	Monoecy	T:C	40	AW39	Monoecy	T:C
16	AW14	Monoecy	C:C	41	AW40	Monoecy	T:C
17	AW16	Monoecy	T:T	42	AW41	Monoecy	T:C
18	AW17	Monoecy	C:C	43	AW42	Monoecy	T:C
19	AW18	Monoecy	T:C	44	AW43	Monoecy	T:C
20	AW19	Monoecy	T:C	45	AW44	Monoecy	C:C
21	AW20	Monoecy	T:C	46	AW46	Monoecy	T:C
22	AW21	Monoecy	T:C	47	AW47	Monoecy	C:C
23	AW22	Monoecy	T:C	48	AW48	Monoecy	C:C
24	AW23	Monoecy	T:C	49	AW49	Monoecy	T:C
25	AW24	Monoecy	T:C	50	AW50	Monoecy	C:C

### Expression analysis of genes in the candidate region

In order to further detect the expression of candidate genes in the fine mapping interval, the qRT-PCR assay was carried out using terminal buds of ‘A36’ and ‘SX.’ The expression of *Bhi08G000338*–*Bhi08G000343* showed no significant difference between ‘A36’ and ‘SX,’ while the expression of *Bhi08G000344* and *Bhi08G000345* was significantly downregulated in ‘SX’ than in ‘A36’ (**Supplemental Figure 2**).

### Phylogenic analysis of CqNET4

**Figure 4:**
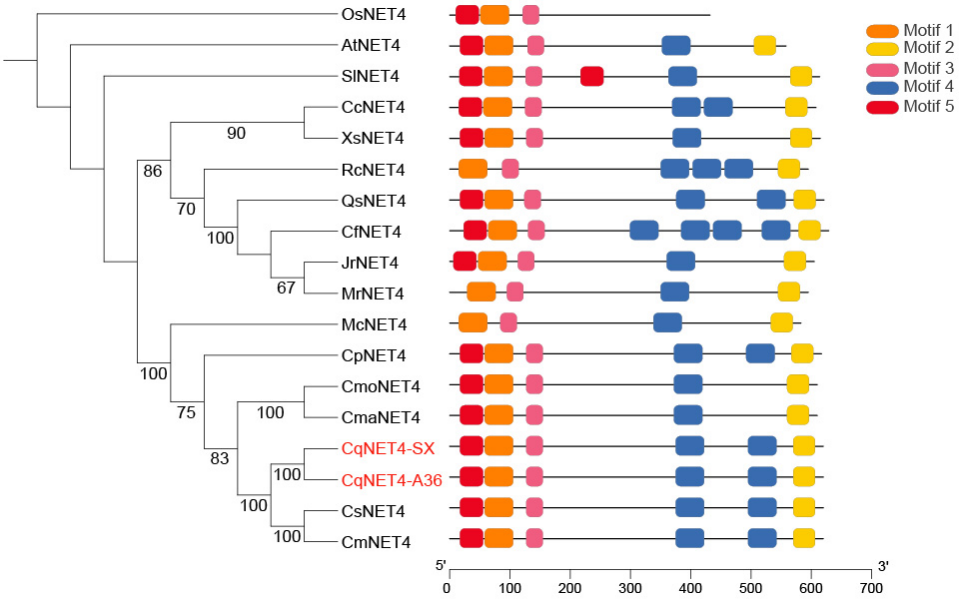
Phylogenetic and motif analyses of NET4A in chieh-qua and other species. The phylogenetic tree was constructed using MEGA-5.2 with 1,000 bootstrap replications. Numbers at the tree forks indicated bootstrap values. The motif distribution of different NET4 from *Oryza sativa* (*Os*), *Arabidopsis* (*At*), *Solanum lycopersicum* (*Sl*), *Cucurbita maxima* (*Cma*), *Cucurbita pepo* (*Cp*), *Myrica rubra* (*Mr*), *Quercus variabilis* (*Qs*), *Xanthoceras sorbifolia* (*Xs*), *Carpinus fangiana* (*Cf*), *Rosa chinensis* (*Rc*), *Clement pomelo* (*Cp*), *Cucumis melo* (*Cm*), *Cucumis sativus* (*Cs*), *Momordica charantia* (*Mc*), and *Cucurbita moschata* (*Cmo*) was shown.

To explore the relationship between CqNET4 and its homologous sequences in other species, we obtained the homolog sequences of CqNET4 using NCBI and TBtools (Chen et al., 2020). The neighbor-joining tree showed that the amino acids in the motif of CqNET4 in plant species were highly conserved (**Supplemental Figure 3**). Moreover, CqNET4 had a close phylogenetic relationship with Cucurbitaceae, including *Cucumis melo* (CmNET4), *Cucumis sativus* (CsNET4), *Cucurbita maxima* (CmaNET4), and *Cucurbita moschata* (CmoNET4) (**Figure 4**). In addition, we found that the NET4 sequences in ‘A36’ and ‘SX’ were longer than those in wax gourd because of a Motif 5 existing in chieh-qua (**Figure 4**).

### Expression analysis of *CqNET4*

**Figure 5:**
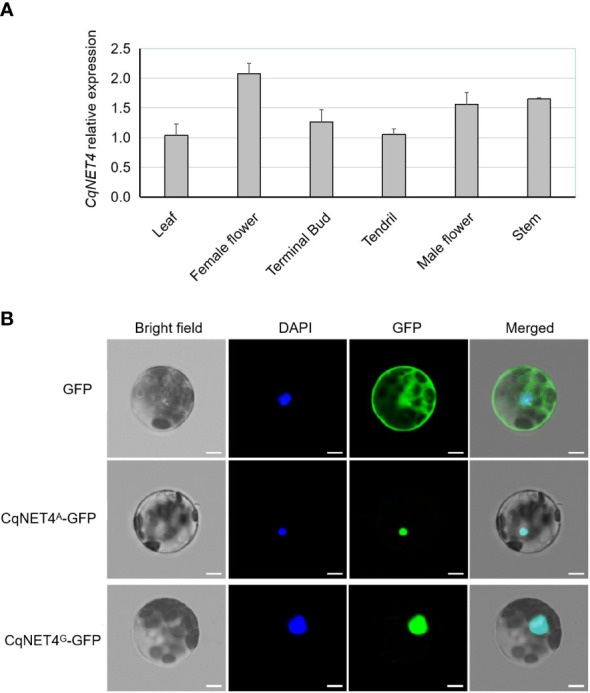
The expression pattern and subcellular localization of CqNET4. **(A)** The relative expression pattern of *CqNET4* in different tissues of chieh-qua. Data are the means ± SE of three independent replicates. **(B)** Subcellular localization of CqNET4^A^ and CqNET4^G^ proteins. Scale bars = 5 µm.

To further understand the *CqNET4* expression pattern in different tissues, we performed the qRT-PCR assay using six chieh-qua tissues, namely, leaves, stems, tendrils, terminal buds, female flowers, and male flowers from SX. Results showed that *CqNET4* was broadly expressed in various tissues, with a high expression in female flowers (**Figure 5A**), implying its crucial role in regulating flower development. Next, to determine the subcellular localization of CqNET4, the full-length CDS sequence without stop codon was cloned and inserted into the pCAMBIA1305 vector. Then, the obtained 35S::CqNET4-GFP fusion construct and 35S::GFP were transiently transformed in the protoplasts of *N. benthamiana* leaf cells. Compared with the GFP control, whose fluorescence signal was distributed throughout the cell, the CqNET4-GFPs fusion proteins of A36 (CqNET4^G^-GFP) and SX (CqNET4^A^-GFP) were both observed exclusively in the nuclei of *N. benthamiana* leaf cell protoplasts (**Figure 5B**), indicating CqNET4 is a nuclear-localized protein and the amino acid change did not influence its subcellular localization.

### Gene expression related to ethylene synthesis

**Figure 6:**
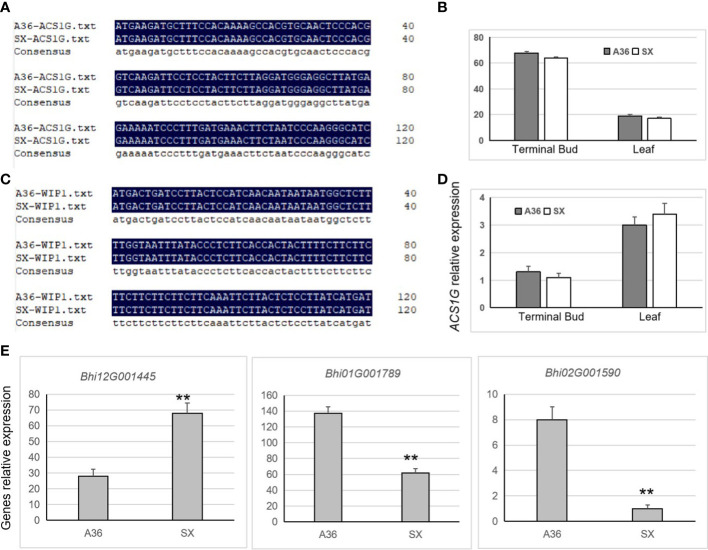
Partial sequence alignment and expression analysis of the homologous genes *WIP1* and *ACS1G* and ethylene synthesis. **(A, B)** Partial sequence alignment results of the homologous genes *WIP1* and *ACS1G* in melon ‘A36’ and ‘SX.’ **(C, D)** Expression levels of *CqACS1* and *CqWIP1* in the leaves and terminal buds of ‘A36’ and ‘SX.’ **(E)** Expression detection of genes related to ethylene synthase. The asterisks indicate significant differences (Student’s *t*-test): *Padj < 0.05, **Padj < 0.01.

*WIP1* and *ACS1G* were identified to control gynoecy development in melon and cucumber, respectively (Boualem et al., 2015; Zhang et al., 2021). To identify whether there existed sequence and expression differences of *WIP1* and *ACS1G* between ‘A36’ and ‘SX,’ we carried out PCR amplification and qRT-PCR assays. Results showed that no differences on the gene sequence of *WIP1* and *ACS1G* were detected between A36 and SX (**Figures 6A, B**). Nevertheless, *WIP1* was not expressed differently between A36 and SX in the terminal bud and leaf tissues (**Figure 6C**), similar to *ACS1G* (**Figure 6D**). Previous studies reported that ethylene played crucial roles in the early stages of floral meristem development of Cucurbitaceae (Boualem et al., 2015; Li et al., 2020; Zhang et al., 2021). Thus, the ethylene synthesis-related genes were chosen for qPCR analysis between the ‘A36’ and ‘SX’ terminal buds. By detecting 18 ethylene synthesis-related genes, only three genes were found significantly changed between ‘A36’ and ‘SX.’ *Bhi12G001445* encoding ACC (1-aminocyclopropane-1-carboxylate) oxidase (ACO) was prominently upregulated in ‘SX’ in comparison with ‘A36.’ Both *Bhi01G001789* and *Bhi02G001590* encoding ACC synthase (ACS) were expressed more highly in ‘A36’ than in ‘SX’ (**Figure 6E**).

## Discussion

Gynoecy could not only improve fruit yield but also reduce labor cost during crossing seed production (Robinson, 1999). Previous studies reported that the gynoecy of bitter gourd was controlled by a single recessive gene (Behera et al., 2009; Cui et al., 2018), as well as that of watermelon (Li et al., 2019) and melon (Boualem et al., 2016). However, in cucumber, some reports showed that its gynoecy was controlled by a dominant gene (Pati et al., 2015) or multiple genes as a quantitative trait (Li et al., 2019). However, as far as we know, no genetic research related to chieh-qua gynoecy has been reported. In this study, by constructing the F_2_ population using gynoecious and monoecious lines, we found that the gynoecy of chieh-qua was controlled by a single recessive locus (**Table 1**), which was consistent with most other cucurbits.

In melon and cucumber, the genetic control for sex determination or female development has been clarified with several crucial genes. Moreover, *WIP1* and *ACS1G* were the crucial genes controlling gyneocy for melon and cucumber, respectively (Boualem et al., 2015; Zhang et al., 2021). Our results showed that *Bhi08G000345* (*CqNET4*) was the most likely possible candidate gene involved in gynoecy in chieh-qua (**Figure 2**) with a higher expression in female flower (**Figure 4A**). Furthermore, we found that CqNET4^G^ was specifically enriched in gynoecious plants, and CqNET4^A^ was in the reference genome B227 and monoecious materials (**Figure 3C**). Thus, we predicted that the chieh-qua gyneocy determination gene was different from other cucurbits.

Microfilament, as part of the cytoskeleton, is involved in important biological processes, including cytoplasmic morphogenesis, signal transduction, and pollen germination and tube growth (Thomas et al., 2009; Blanchoin et al., 2014). NET4s are plant microfilament-specific binding proteins, which potentially couple different membranes to the actin cytoskeleton in plant cells (Deeks et al., 2012). Overexpression of *NET4A* leads to a shorter root length in *A. thaliana* (Kaiser et al., 2019). NET2 can interact with pollen tube receptor kinase to co-regulate pollen tube development (Duckney et al., 2017). The dual mutation of *NET3B* and *NET3C* could result in a lethal phenotype of *Arabidopsis* gametophytes (Wang et al., 2014). Overexpression of *AtNET3B* could enhance the association between ER (endoplasmic reticulum) and the actin cytoskeleton (Wang and Hussey, 2017). A further study indicated that NET4 is localized to highly constricted regions of the vacuolar membrane in *A. thaliana* (Kaiser et al., 2019). However, our study found that CqNET4^A^ and CqNET4^G^ proteins were located in the cell nucleus, which was different from previous studies. Thus, we predicted that the CqNET4 protein might have homology with other species, while it possessed a novel function during the chieh-qua sex determination.

Previous studies demonstrated that the phytohormone ethylene played critical functions in controlling female or male flower development (Wang et al., 2010b; Manzano et al., 2014; Boualem et al., 2015; Martínez and Jamilena, 2021; Zhang et al., 2021). The gyneocy crucial gene *WIP1* functions in a pathway with other ethylene-related genes such as *CmACS11* and *CmACS-7* in melon (Boualem et al., 2015). *CsACS1G* (a vital enzyme for ethylene synthesis) is the critical gene responsible for the formation of female flowers, rather than *CsACS1* or other genes in the *F* locus (Zhang et al., 2021). Our results showed that the sequence and expression level of homologous *WIP1* and *ACS1G* between A36 and SX showed no difference (**Figures 6A–D**). In addition, the ethylene synthesis-related genes encoding ACS were significantly upregulated in gyneocious plants (**Figure 6E**). Therefore, we propose that *CqNET4* might regulate chieh-qua gynoecy by affecting ethylene synthesis. However, the biological function of *CqNET4* should be elucidated by combining efficient genetic transformation systems such as genome editing or overexpression technology in the future.

## Conclusion

Our study found that the gynoecy of chieh-qua was controlled by a single recessive gene, and it was fine-mapped to a 530-kb region between Indel-3 and KASP145 on Chr.8. Combining the mapped cloning, sequence, and expression results, we predicted *Bhi08G000345* (*CqNET4*) as the candidate gene of gynoecy, which differed from the homology of gynoecious genes, such as *WIP1* or *ACS1G* in melon and cucumber.

## Data availability statement

The datasets presented in this study can be found in online repositories. The names of the repository/repositories and accession number(s) can be found in the article/**Supplementary Material**.

## Author contributions

Conceived and designed all the experiments: MW, SY, and QP. Performed part of the experiments: WeiL, ZC, and JY. Analyzed the data: LC, WenL, and DX. Wrote the manuscript: MW. Edited the manuscript: BJ. All authors read and approved the final manuscript.
