# Supplementary figures and images for: Fine mapping and candidate gene analysis of gynoecy trait in chieh-qua (Benincasa hispida Cogn. var. chieh-qua How)
Source: Front Plant Sci. 2023 Apr 20;14:1158735. doi: 10.3389/fpls.2023.1158735 (PMC10157166; doi:10.3389/fpls.2023.1158735)

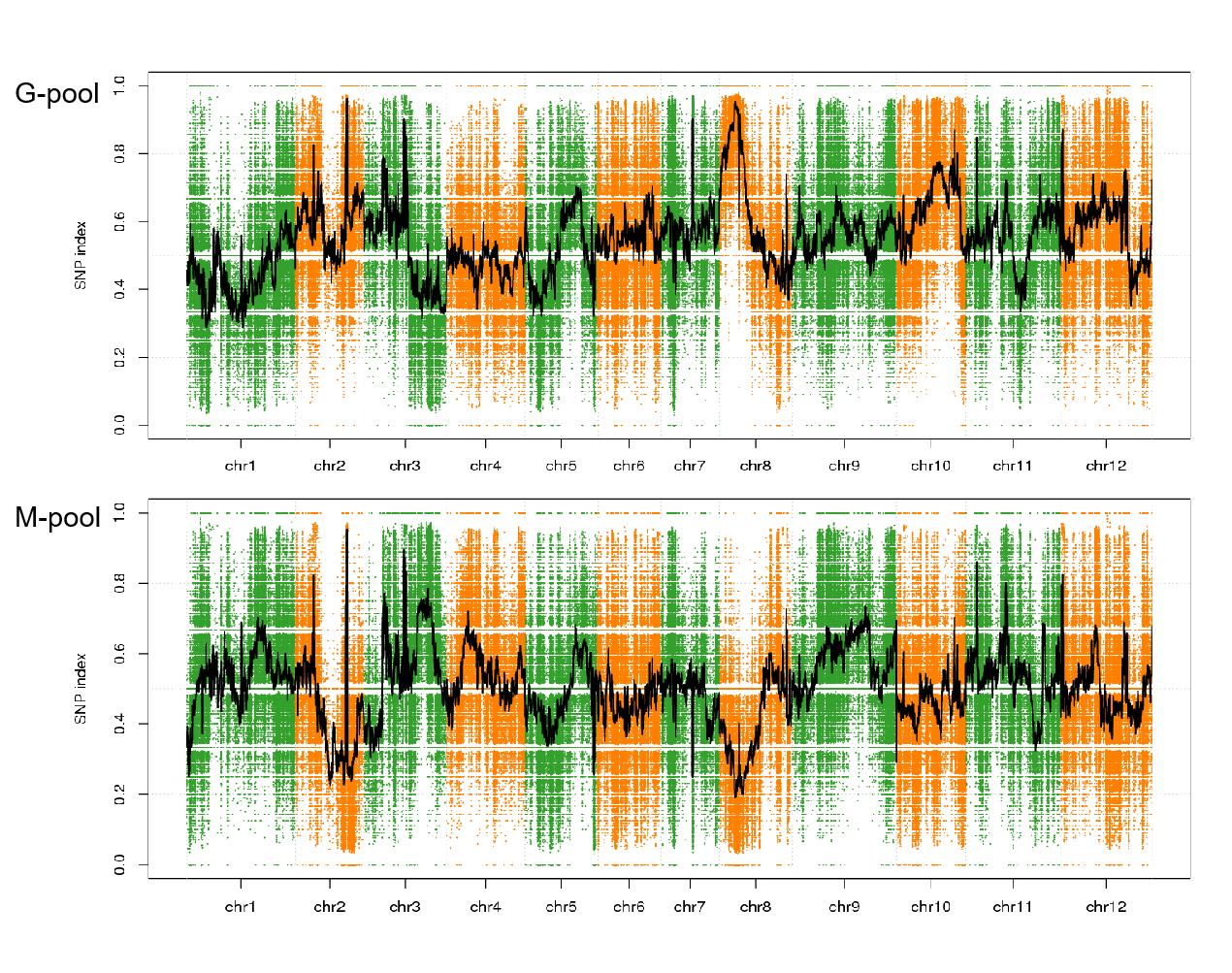

Supplement: Supplementary file 1 [file Image_1.jpeg]

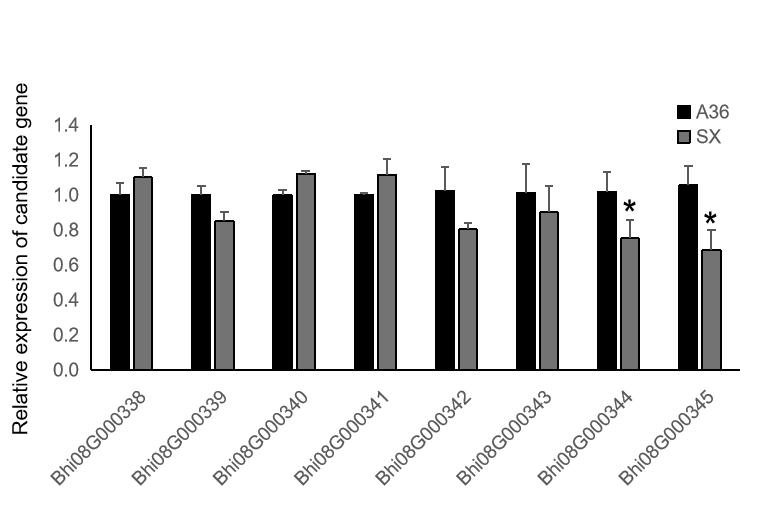

Supplement: Supplementary file 2 [file Image_2.jpeg]

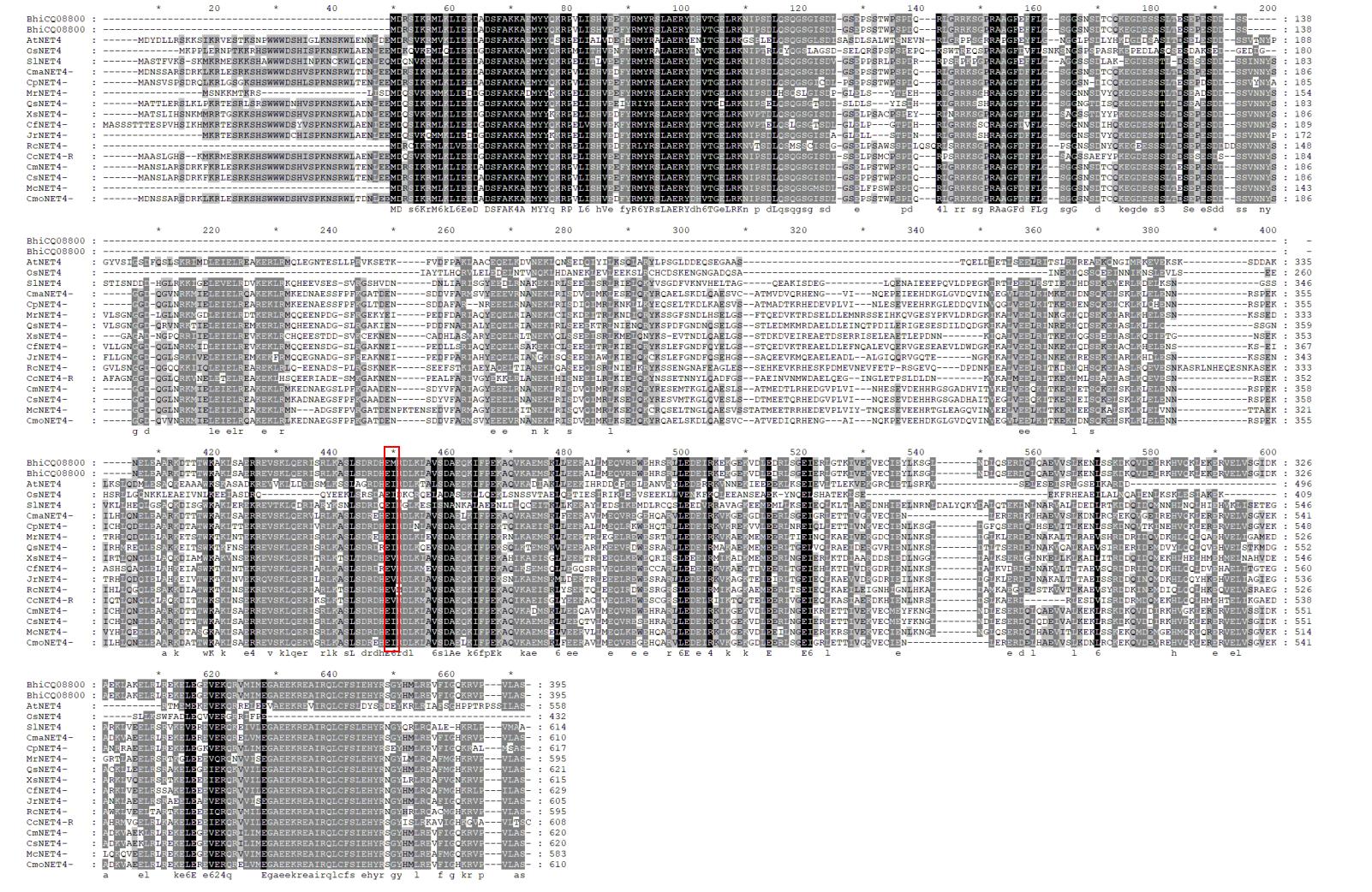

Supplement: Supplementary file 3 [file Image_3.jpeg]
